# Pressure-driven phase transitions and reduction of dimensionality in 2D silicon nanosheets

**DOI:** 10.1038/s41467-018-07832-4

**Published:** 2018-12-21

**Authors:** Gil Chan Hwang, Douglas A. Blom, Thomas Vogt, Jaejun Lee, Heon-Jin Choi, Sen Shao, Yanming Ma, Yongjae Lee

**Affiliations:** 1 Department of Earth System Sciences, Yonsei University, Seoul, 03722 Korea; 2 NanoCenter & Department of Chemical Engineering, University of South Carolina, Columbia, SC 29208 USA; 3 NanoCenter & Department of Chemistry & Biochemistry, University of South Carolina, Columbia, SC 29208 USA; 4 Department of Materials Science and Engineering, Yonsei University, Seoul, 03722 Korea; 5 State Key Lab of Superhard Materials & Innovation Center for Computational Physics Methods and softwares, College of Physics, Jilin University, 130012 Changchun, China; 6 International Center of Future Science, Jilin University, 130012 Changchun, China; 7 Center for High Pressure Science and Technology Advanced Research, 201203 Shanghai, China

## Abstract

In-situ high-pressure synchrotron X-ray powder diffraction studies up to 21 GPa of CVD-grown silicon 2D-nanosheets establish that the structural phase transitions depend on size and shape. For sizes between 9.3(7) nm and 15.2(8) nm we observe an irreversible phase transition sequence from I (cubic) → II (tetragonal) → V (hexagonal) during pressure increase and during decompression below 8 GPa the emergence of an X-ray amorphous phase. High-angle annular dark field scanning transmission electron microscopy (HAADF-STEM) and atomic force microscopy (AFM) images of this X-ray amorphous phase reveal the formation of significant numbers of 1D nanowires with aspect ratios > 10, which are twinned and grow along the <111> direction. We discovered a reduction of dimensionality under pressure from a 2D morphology to a 1D wire in a material with a diamond structure. MD simulations indicate the reduction of thermal conductivity in such nanowires.

## Abstract (web summary)

Silicon nanostructures have important applications as functional materials. Here the authors investigate the structural transformations of 2D silicon nanosheets under compression and decompression, showing the irreversible formation of 1D silicon nanowires.

## Introduction

Silicon is an abundant, low-cost, non-toxic, environmentally friendly, biocompatible, and ubiquitous chemical element used in our daily lives and industrial applications^1^. At ambient conditions it crystallizes in a cubic diamond structure with space group *Fd*${\bar{3}}$*m*, and its structural behavior with temperature^2–7^, coefficients of thermal expansion^4,8–10^ and negative thermal expansion (NTE) at low temperatures^11,12^ have been well established. Changes of silicon’s crystal structure at high pressures revealed metallic structures with Si in 6-fold, 8-fold, and 12-fold coordination^13^. Various allotropes including framework structures have been studied in recent years^14^. Amorphous, semiconducting silicon (*a-Si*) made by chemical or physical vapor deposition is generally believed to have a four-coordinated, non-periodic continuous random network (CRN) with 5-membered, 6-membered, and 7-membered rings of tetrahedra. Introduced by Zachariasen^15^, *a-Si* is metastable with respect to crystalline silicon which has only 6-membered rings. Reduced density functions of CRN models made using bond-swapping algorithms^16,17^ and framework relaxation^18,19^ match experimental high-energy X-ray, neutron^20^ and electron diffraction data^21^. However, as pointed out by Treacy and Borisenko^22^, reduced density functions cannot unequivocally distinguish between different CRN models. Other models for *a-Si* structures are the paracrystalline model of Hosemann and Baggchi^23^ and the microcrystallite model of Turnbull and Polk^24^ which posit that short-range ordered material at length scales of a few nanometers are subjected mainly to strain gradients in the paracrystalline model or rotational disorder in the microcrystallite model. Fluctuation electron microscopy reveals the existence of 1–2 nm regions of high crystallinity in different samples referred to as *a*-Si^25–27^. The short-range order at the nanometer scale found in diffraction experiments of many nanomaterials results in them being labeled “X-ray amorphous”. However, electron microscopy and in particular high-angle annular dark field scanning transmission electron microscopy (HAADF-STEM) has established the presence of well-ordered structural regions with short-range translational symmetry^28^. This motivates HAADF-STEM investigations of highly disordered materials found after high-pressure X-ray diffraction experiments which often reveal few or no sharp X-ray and/or neutron diffraction peaks.

The structures and properties of distinct silicon nanomaterials such as zero-dimensional, one-dimensional, and two-dimensional quantum dots, nanowires and nanosheets (NS) are well established^29,30^. 2D NS materials have thicknesses less than 2 nm and reveal <110> zone axes, often with edges of defects oriented along <111>^31^. We explored the high-pressure chemistry of 2D Si NS and found that the phase transition pressures depend on the average crystallite size and shape. We observe that below 8 GPa during decompression large amounts of 2D-materials with crystallite sizes smaller than 15 nm transform into x-ray amorphous materials which when imaged using STEM after pressure release contain large amounts of 1D nanowires. Si nanowires are an important class of nanomaterials used as biosensors for the detection of metal ions, nucleic acids, and viruses^32^. Their dimensionality strongly impacts properties such as thermal conductivity^33,34^.

Fabrication and synthesis of 1D Si nanowires is achieved using top-down approaches such as electron-beam or nanoprint lithography or bottom-up techniques based on vapor-liquid-solid (VLS) or oxide-assisted growth^35^. The contamination of Au used as a catalyst in VLS synthesis^36^ in Si nanowires, the presence of SiOx layers after oxide-assisted growth as well as a better control of the aspect ratio, twinning, and defects are current challenges for Si 1D-nanowire synthesis. We believe that the pressure-driven reduction in dimensionality of nanomaterials established here for silicon 2D-nanosheets made by chemical vapor deposition (CVD) is a fabrication method that should lead to more high-pressure investigations of other 2D nanomaterials as it opens a route to high purity materials with many potential applications in sensing, thermal electric devices and solar energy conversion^37^.

## Results and Discussion

### Ambient conditions

**Fig. 1:**
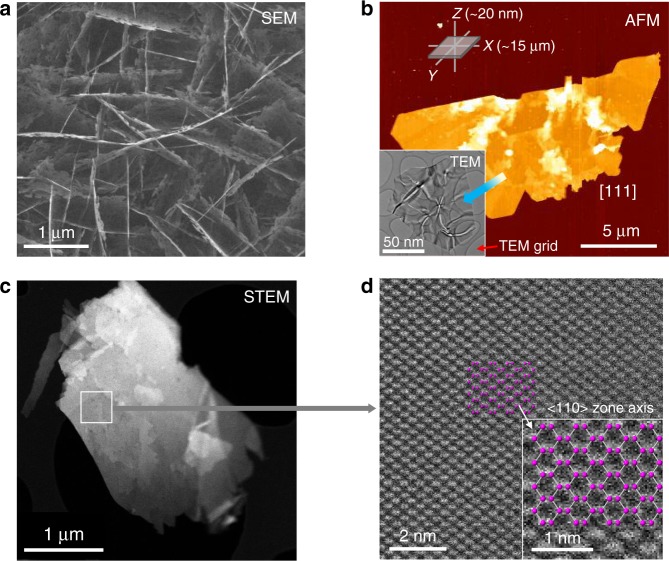
Images of silicon nanosheets. **a** SEM image of a silicon nanosheet (Si-NS). **b** AFM, and TEM images of the silicon particle of [111] direction, about 20 nm thick. **c**, **d** STEM images of Si-NSs showing surfaces with silicon dumbbells along the <110> zone axis

**Fig. 2:**
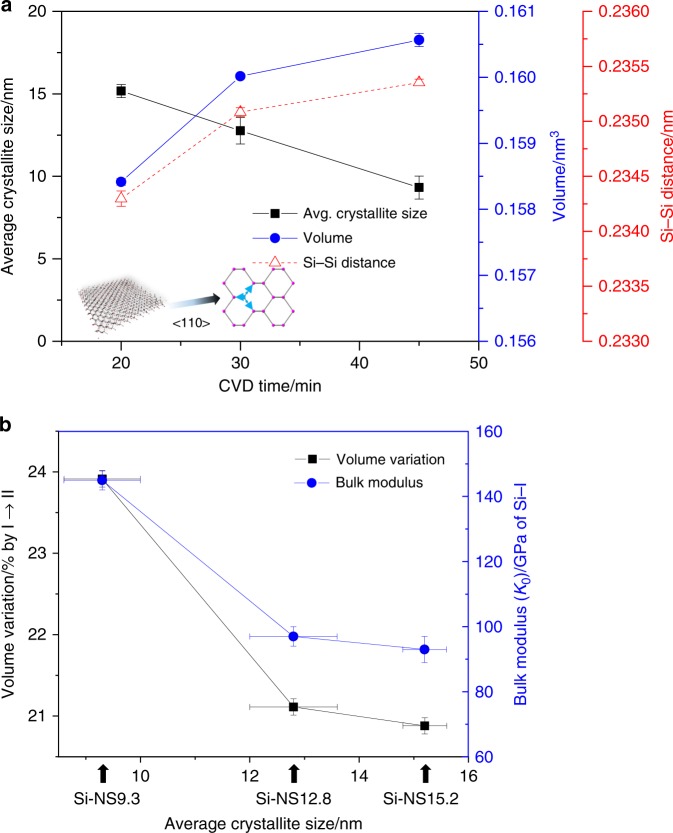
Physical parameters and pressure response of Si-nanosheets. **a** Crystallite size and unit cell volume as a function of CVD reaction time. Using the peak width of the (111) reflection, crystallite sizes were derived using the Scherrer equation. **b** Bulk moduli of nanoscale Si-I phase and the degree of volume contraction upon Si-II transition as a function of crystallite size (arrows)

As synthesized silicon nanosheets were characterized by SEM, AFM, TEM, and STEM as shown in Fig. 1. The SEM and AFM images in Fig. 1a, b confirm the 2D nature of the as-grown materials. The HAADF STEM data in Fig. 1c, d show the typical dumbbell structure of the diamond cubic Si phase in a <110> zone axis orientation. The majority of the as-synthesized silicon nanosheets (Si NS) were found to have a <110> orientation with extended defects along <111>. Unit-cell parameters of the silicon nanoscale materials at ambient condition were derived using XRD data measured on samples mounted inside quartz capillaries (Ø0.5 mm) (Supplementary Fig. 1, Supplementary Table 1). The (111), (220), (311), (400), and (331) Bragg peaks up to 48° in 2*θ* (Mo-Kα_1_, *λ* = 0.070932(1) nm) (Supplementary Fig. 1) were used to determine the unit-cell volumes to be 0.1584(1), 0.1600(1), and 0.1606(1) nm^3^, for the Si-NS15.2, Si-NS12.8, and Si-NS9.3 samples, respectively. Using the peak width of the (111) reflection, respective crystallite sizes were derived using the Scherrer equation^38^. The crystallite sizes for the Si-NS15.2, Si-NS12.8, and Si-NS9.3 samples were 15.2(4), 12.8(8), and 9.3(7) nm, respectively (Fig. 2a, Supplementary Fig. 1). It appears that there is a minimum in crystallite size and a concomitant maximum in unit cell volume when the CVD reaction time is 45 min (Fig. 2a, Supplementary Table 1).

### Pressure-induced phase transition and recovery of X-ray amorphous silicon

In bulk silicon, the transition sequence during compression has been reported to be I → II → V → VI → VII up to about 50 GPa^39^, whereas during decompression the transitions found were V → (V + II) → II → (II + III) → III^40,41^. Depending on the decompression speed the recovered phases were either phase I^42^, III, XI^42^, or an X-ray amorphous phase^43^. The high-pressure VI, VII, and X phases are observed at pressures larger than 34 GPa^44^. In particular, phase X (*fcc*) is only stable between 78.3 and 230 GPa^45,46^.

We performed high-pressure diffractions experiments up to 20.5(1), 21.0(1), and 20.5(1) GPa for the Si-NS9.3, Si-NS12.8, and Si-NS15.2 samples, respectively. The high-pressure silicon phases have previously been established as Si-I (*Fd*${\bar{3}}$*m*, cubic, *Z* = 8), Si-II (*I*4_1_/*amd*, tetragonal, *Z* = 4), Si-V (*P*6/*mmm*, hexagonal, *Z* = 1), and X-ray amorphous silicon (*a*-Si). The observed phase transition sequence of the 2D silicon nanosheet samples is I → II → V under compression and V → II → *a*-Si during decompression for Si-NS9.3, Si-NS12.8, and Si-NS15.2. In contrast to bulk silicon samples, we did not detect any evidence for the presence of the Kasper phase (Si-III) and lonsdaleite (Si-IV) at pressures up to 9 GPa^47–49^. The well-established (111) twinning in silicon has an ABAB stacking sequence and can be understood as a fragment of lonsdaleite (Si-IV). The orientation relationships (111)_Si-I_ || (001)_Si-IV_ || (100)_Si-III_ were established in electron microscopy studies^49^. However, larger domain sizes which would also diffract in an X-ray powder diffraction experiment require the transfer of the hexagonal motif from a template such as a hexagonal GaP nanowire upon which one can then epitaxially grow an extended Si shell^50^.

**Fig. 3:**
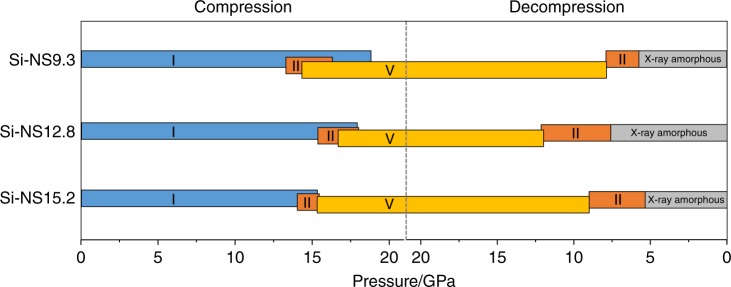
Transition sequence of silicon nanosheets. Phase transitions for Si-NS9.3, Si-NS12.8, and Si-NS15.2 during compression up to 20.5(1), 21.0(1), and 20.5(1) GPa, respectively, and then decompression

The high-pressure phase transitions for the Si-NS9.3, Si-NS12.8, and Si-NS15.2 samples are irreversible and in all cases X-ray amorphous *a*-Si is found below 8 GPa during decompression. The onset pressure of individual phase transitions, the region where two phases coexist, and the hysteresis upon decompression differ slightly but not systematically and are summarized in Fig. 3 (Supplementary Table 2 and Supplementary Figs. 2–4).

The Si–Si distance and hence the unit cell volume of Si NS vary in response to pressure^51–53^. The detailed pressure-dependent changes of the normalized unit cell volumes for the Si-NS samples given in Supplementary Figs. 2b–4b and the bulk moduli of various phases point to the rich high-pressure chemistry and its dependence on crystallite size and shape. During decompression from 19.0(1) GPa, the formation of *a*-Si using porous silicon was first reported by Deb et al.^54^ and pressure-induced amorphization of Si following a compression/decompression cycle was subsequently investigated^55,56^. Using wafer indentation experiments, Gogotsi et al. studied the phase transitions of silicon as a function of decompression speed and observed that silicon transformed to an amorphous phase near 3 GPa, below which it reverted to Si-I or Si-III^57,58^. Recent theoretical work predicted that metastable silicon following decompression would transform to Si-III^59^. We have confirmed by STEM imaging after 12 months that these nanowires are stable after pressure release and no reversible phase transition back to 2D-nanosheets occurs. The recovered X-ray amorphous silicon phases after compressing and decompressing silicon 2D-NS are therefore inconsistent with results on bulk Si and point to the important role of dimensionality and size of 2D-Si under pressure.

**Fig. 4:**
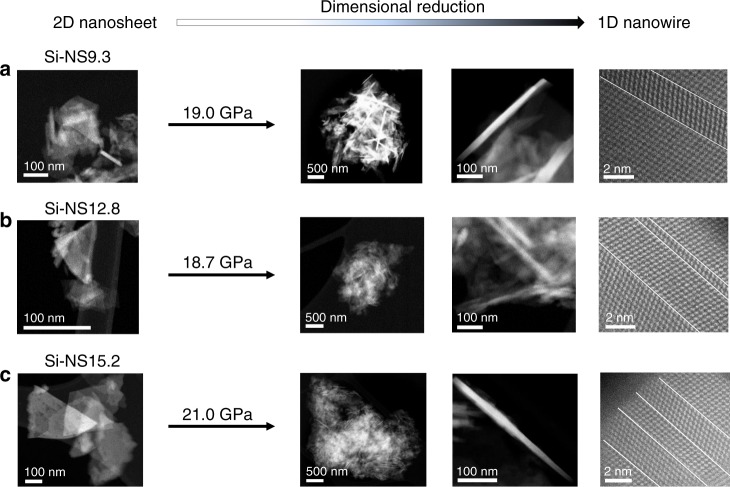
HAADF-STEM images of Si-nanowires formed from Si-nanosheets. 2D-Si-nanosheets of different crystallite sizes are seen on the left. **a** Si-NS9.3. **b** Si-NS12.8. **c** Si-NS15.2 Increasing magnification HAADF-STEM images of nanowires found in X-ray amorphous materials subsequent to high-pressure and complete decompression. White lines are the twinning planes {111} for Si. The 2D Si-nanosheets transformed to 1D Si-nanowires during decompression at room temperature

**Fig. 5:**
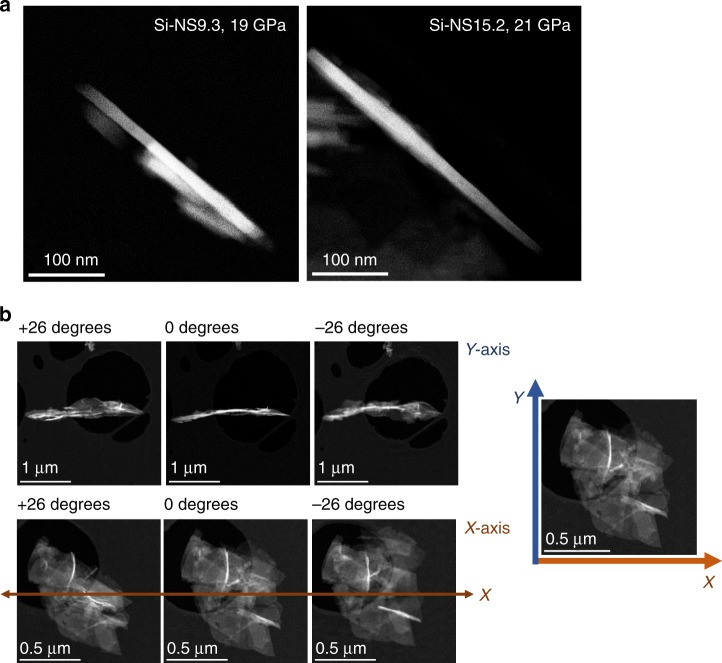
HAADF-STEM images of isolated 1D Si-nanowires. **a** Isolated Si-NS9.3_19 GPa and Si-NS15.2_21 GPa. **b** The rotational HAADF-STEM images of the 1D Si-nanowires

HAADF STEM images from the X-ray amorphous materials in Fig. 4 all reveal significant amounts of well-ordered 1D nanowires (see also rotational STEM images in Fig. 5). HAADF STEM images of the starting materials are shown in the left most column of Fig. 4 for reference. After being subjected to high pressure and decompression below 8 GPa, the low magnification HAADF STEM images in the second column indicate the amount of 1-D nanowires formed. The long axis of the nanowires is along <111> and the nanowires are typically in a <110> zone axis orientation relative to the electron beam. Significant twinning of the nanowires along <111> can be seen in the far right column of Fig. 4 for all the Si NS samples. It appears that under decompression the defects of the 2D-Si nanosheets which predominantly were located on edges pointing in the <111> direction grew to become the long axis of the 1D nanowires. We found aspect ratios above 50 with observed widths of about 15 nm and lengths near one micron. This is in the size range of what can be done using top-down wafer-scale patterning^60^. Our samples obtained from the gaskets after high-pressure X-ray experiments (Fig. 4) were not sonicated nor were any other attempts made to further isolate and separate the Si nanowires. The only important step was to dissolve the silicon oil used as a pressure-transmitting medium as it degrades in the electron beam. We observed regions on our microscopy grid where 1D-Si nanowires are well separated from the bulk of the material, which will become advantageous when using micromanipulators. Important future work would be to attempt to replicate our work done in a diamond anvil cell using nano-indentation.

**Fig. 6:**
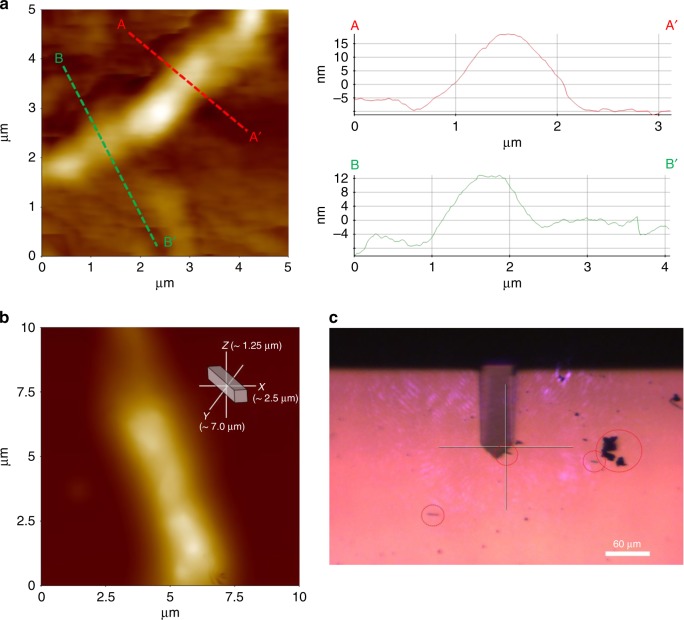
AFM images of isolated 1D Si-nanowires. **a** Si-NS9.3_21 GPa and its 1-dimensional height profile sections. **b** Another AFM image of Si-NS9.3_21 GPa with its dimension. **c** Optical image of nanowires (circled) dispersed on a glass substrate (Park Systems, XE-100)

It has been shown that nano-indentation can transform crystalline Si in wafers into various high-pressure polymorphs and, after decompression, into an amorphous Si phase^61^. Aberration-corrected STEM offers unique opportunities to characterize and distinguish “X-ray amorphous” phases as it does not rely on the presence of materials with extended translation symmetry. Our work points to a route for top-down pressure-driven nanowire synthesis using 2D-Si NS which might be applicable to other nanomaterials. The pressure-driven formation of the isolated Si nanowires has also been confirmed by AFM imaging (Fig. 6). Our unequivocal detection of high-density 1D Si nanowires in these X-ray amorphous phases made by compressing 2D nanosheets establishes a synthesis process for materials with applications in solid-state electronics and photovoltaics technologies.

### Molecular dynamics calculations of thermal conductivity

**Fig. 7:**
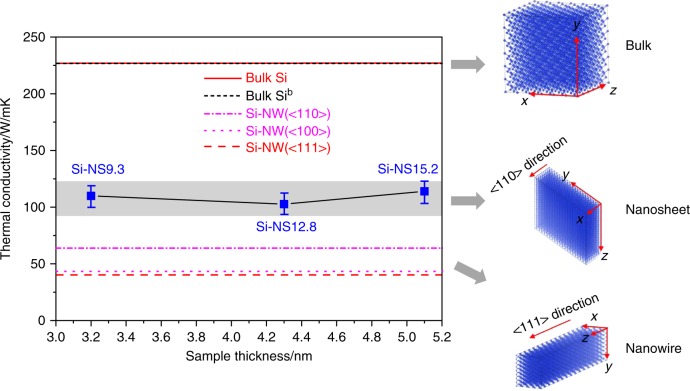
Calculated thermal conductivity of bulk silicon and Si-nanostructures. The thermal conductivity of bulk Si, bulk Si^b^ (predicted by Zhou^62^), <111> direction Si-NW, <110> surface Si-NW (predicted by Zhou^62^), <100> direction Si-NW (predicted by Donadio^63^), Si-NS9.3, Si-NS12.8, and Si-NS15.2. Gray area is the fluctuant range of the thermal conductivity for nanosheets. The model systems studied in this work for bulk silicon, silicon nanosheet with <110> zone axis orientation, and silicon nanowire of which the large dimension is along <111> direction, are shown as insets

One physical consequence of reducing the dimensionality to 1D-Si nanowires is a reduction of the thermal conductivity along the direction of the wire. The calculated thermal conductivity of bulk silicon is 226.54 ± 18.17 W/mK and in good agreement with the value of 226.07 ± 2.86 W/mK given by Zhou^62^ using the same potential and method. As shown in Fig. 7, the thickness of the 2D silicon nanosheets has a minute effect on the thermal conductivity resulting in an average value of 106 W/mK, while the thermal conductivity along the <100> direction for a Si-NW was found to be 39.62 ± 4.51 W/mK. Previous theoretical work revealed that the thermal conductivities along the <110>^62^ and <100> direction^63^ for Si-NW are 64.5 ± 4.5 and 43 ± 10 W/mK, respectively. The thermal conductivities of Si-NWs are significantly below that of Si-NS and bulk materials, indicating that the thermal conductivity reduces significantly with the reduction of dimensionality. This reduction of dimensionality is a promising way to increase the ZT coefficient which is a dimensionless figure of merit defined as ZT = *S*^2^*Tσ/κ*, with *S* being the Seebeck coefficient, *κ* the thermal conductivity, and *σ* the electrical conductivity at temperature *T*. Therefore, silicon nanomaterials and other nanowires with a diamond structure made by using pressure have the potential to be applied to improved thermoelectrics.

## Methods

### Sample synthesis

The synthetic silicon nanosheet samples used in this study were prepared by Kim et al.^31^ A *c*-plane sapphire substrate was used in CVD to produce Si-NS12.8 and Si-NS15.2 nanosheets while a quartz substrate was used for Si-NS9.3. In this paper the sample name is given by NS (nanosheet) followed by the average crystallite in nanometer. The sapphire substrate was first subjected to a wet cleaning process and then placed in the center of a furnace through which Ar and H_2_ flowed at 300 sccm (standard cubic centimeters per minute) as the temperature was increased to 1273 K over a period of 25 min. Once the temperature reached 1273 K, the flow rate was increased to 3000 sccm. Pure samples of Si-NS15.2, Si-NS12.8, Si-NS9.3 were obtained by bubbling H_2_ at 20 sccm in liquefied SiCl_4_ for 20 min, 30 min, 45 min, respectively^31^.

### Electron imaging

We identified the crystallite types of 15 µm long and 20 nm thick samples using field emission scanning electron microscopy (FE-SEM), atomic force microscope (AFM), and transmission electron microscopy (TEM). The sheet size varied depending on the H_2_ bubbling time and/or amount. The surface was either the [111] or [110] plane depending on the synthesis conditions (Fig. 1)^31^. An AFM image using a Nanowizard I (JPK instruments) with tapping mode was obtained. Abberation-corrected STEM (JEOL JEM-2100F) was performed at 200 kV in the high angle annular dark field mode. Imaging with an SEM (JEOL JSM-7100F) was done at 15 kV (×10,000) without coating.

### Phase identification and crystallite size

X-ray powder diffraction was performed in transmission method to qualitatively analyze each 2D-Si-NS sample using a MicroMax-007HF (Rigaku Corp.) equipped with a rotating anode Mo-Kα_1_ (*λ* = 0.070932(1) nm) with multilayer optics (VariMax-Mo, Rigaku) and an imaging plate (IP) detector (*R*-axis IV^+^^+^, Rigaku). The X-ray generator was operated at 1.2 kW (50 kV, 24 mA), and the X-ray beam size was reduced to 100 µm by using a pin-hole collimator. The sample-to-detector distance was 120 mm and the typical exposure time was 10 min. Samples were rotated between 0 and 180° using a quartz capillary with a diameter of 0.5 mm. IPanalyzer v3.551 and Crystalclear v2.0 programs were used to calibrate and process the 2D diffraction images^64,65^. LaB_6_ (NIST SRM 660c, *a* = 0.4157044(8) nm) standard powder was used for the instrument calibration and initial condition.

The crystallite size (*D*) was calculated using the Scherrer equation, *D*_(111)_ (nm) = 0.94*λ*/*β* cos*θ*^38^, where *λ* is X-ray wavelength, *θ* (radian) is diffraction angle, and *β* (radian) is *β*_sample broadening_-*β*_instrumental broadening (SRM LaB6 660c, FWHM = 0.2042 nm)_ at the full width at half maximum (FWHM) which was calculated using profile-fitting method with a pseudo-Voigt function in the CMPR software^66^. The Scherrer constant used was 0.94 for cubic symmetry. The peak broadening was measured using a Si standard and the same instrumental conditions. We would like to point out that the crystallite sizes obtained from diffraction measurements and those observed in STEM images are based on completely different physical mechanisms^67^ and in most cases will not agree. However, in-situ crystallite determinations in a diamond anvil cell provide us with a qualitative guidance of the crystallite size which we cannot be obtained using imaging methods.

### High-pressure experiments

High-pressure X-ray diffraction experiments were performed at the Pohang Accelerator Laboratory (PAL). The Si-NS12.8 and Si-NS15.2 samples were measured at the PLS-II 9 A beamline at PAL. Passing a double crystal monochromator equipped with bent Si (111) and Si (311) crystals and a pinhole, monochromatic X-rays with a wavelength of 0.0622(1) nm were used. The angle-dispersive X-ray diffraction data were measured using a CCD detector (Rayonix SX165, 2048 × 2048 pixels, exposure time of 150 s). The Si-NS9.3 sample was measured at the PLS-II 5 A beamline at PAL. Monochromatic X-ray with a wavelength of 0.0692(1) nm was used in combination with an image plate detector (marXperts mar345, 3000 × 3000 pixels, exposure time ~150 s). A LaB_6_ (NIST SRM 660c, *a* = 0.4157(1) nm) standard powder was used for the instrument calibration.

As a high-pressure device we used a symmetric-type diamond-anvil cell (SDAC). The culet diameter of the diamond anvil (type I) was approximately 400 μm. Stainless steel T301 (thickness 0.25 mm) was used as a gasket material after indentation to about 80 μm. The sample chamber in the gasket was formed using an electric discharge machine (EDM; Holozoic products) and was about 150 μm in diameter. The pressure-transmitting medium used to generate hydrostatic conditions on the sample was a methanol: ethanol: water mixture (16:3:1 by volume) in the case of Si-NS12.8 and Si-NS15.2, and silicone oil (10 cSt) in the case of Si-NS9.3^68,69^. The pressure was increased from ambient to ca. 8.0 GPa in ca. 0.5 GPa increments at room temperature. The sample was equilibrated at each pressure for about 15 min before the XRD measurement. The pressure was calculated from the shift of the ruby (>40 μm in size) R1 emission line and the established formulae of *P* (±0.05 GPa) = 1904/7.665 [(1 + (Δ*λ*/*λ*_0_))^7.665^-1]^70^. The lattice parameters were refined by applying the whole-pattern fitting LeBail method using the GSAS suite of programs^71,72^. Bulk moduli from normalized volume (*V/V*_0_) were calculated using the Murnaghan equation of state (*V*/*V*_0_ = [1 + *K*´*P*/*K*_0_]^-1/^^*K'*^), *K*´ = (Δ*K*/Δ*P*)_*P* = 0_ = 4) or *P* = 3/2*K*_0_[(*V*/*V*_0_)^-7/3^-(*V*/*V*_0_)^-5/3^](1 + 3/4(*K*_0_´-4)[(*V*/*V*_0_)^-2/3^-1]), *K*_0_ = -*V*(Δ*P*/Δ*V*)_*P* = 0_, *K*_0_´ = (Δ*K*/Δ*P*)_*P* = 0_^73,74^, which was integrated within the EOSFIT and the OriginLab programs^75,76^.

### Scanning transmission electron microscopy

The HAADF-STEM images of materials before after compression were taken using a JEOL JEM 2100F equipped with a CEOS-corrector to minimize spherical aberration of the electron probe. The powder samples were meticulously and painstakingly cleaned with hexane to remove the silicone oil used as a pressure-transmission fluid in the X-ray experiments before Z-contrast imaging. Failure to do so will lead to rapid decomposition of the silicone oil and subsequently the samples will degrade under the 200 kV electron beam. Cleaned and dried powders were loaded onto holey carbon-coated copper grids. The electron probe used at 200 kV to image the sample had a 24 mrad convergence angle. The images were recorded with a Fischione Model 3000 detector and a camera length such that the detector spanned between 75 and 178 mrad. The scanning acquisition was synchronized to the 60 Hz AC electrical power to minimize 60 Hz noise in the images. A pixel dwell time of 15.8 μs was used.

### Molecular dynamics simulation

All theoretical MD simulations were performed using the Tersoff potential^77^ for interatomic interactions in an equilibrium molecular dynamics (EMD) calculation based on the Green-Kubo formula^78^ in a large-scale atomic/molecular massively parallel simulator environment (LAMMPS)^79^. The model system we studied in this work is shown in Fig. 7. We chose a simulation cell of Lx × Ly × Lz = 3.265 × 3.265 × 3.265 nm^3^ for bulk silicon. In the nanosheet simulations, the thicknesses along the *x* direction were set to 3.2 nm, 4.3 nm, and 5.1 nm, for the samples NS9.3, NS12.8, and NS15.2, respectively, and the areas perpendicular to the *x* direction are used for the same size of Ly × Lz = 11.54 × 11.42 nm^2^. We employ free boundary condition in the *x* direction and periodic boundary conditions in the y and z directions. For the Si nanowire (Si-NW), the cross-section (*xy* plane) is chosen as Lx × Ly = 5.395 × 5.535 nm^2^ and free boundary condition is used along the *x* and *y* directions. Furthermore, periodic boundary conditions are employed along the *z* direction and the length of Lz was chosen to be 23.56 nm. A time step of 1 fs was employed in our simulations. The bulk and 2D-nanosheets are relaxed in the NVT ensemble at 300 K for 500 ps to obtain the optimized structures, and then the heat current is calculated in the NVE ensemble. A longer 7 ns run is needed for Si-NW in the NVT ensemble to reach the equilibrium structure. NVT (constant crystallite, constant volume, and constant temperature) is a canonical ensemble. NVE (constant crystallites, constant volume, and constant total energy) is a micro-canonical ensemble.

## Supplementary information

Supplementary Information

## Data Availability

The data that support the findings of this study are available within the article and its Supplementary Information file or available from the corresponding author upon reasonable request.
