# Supplementary material for: Pressure-driven phase transitions and reduction of dimensionality in 2D silicon nanosheets
Source: Nat Commun. 2018 Dec 21;9:5412. doi: 10.1038/s41467-018-07832-4 (PMC6303324; doi:10.1038/s41467-018-07832-4)
Supplement: Supplementary file 1 — Supplementary Information [file 41467_2018_7832_MOESM1_ESM.pdf]

Supplementary Information

Pressure-Driven Phase Transitions and Reduction of Dimensionality in 2D Silicon Nanosheets

Gil Chan Hwang[1], Douglas A. Blom[2], Thomas Vogt[3], Jaejun Lee[4], Heon-Jin Choi[4], Sen Shao[5], Yanming Ma[5,6], Yongjae Lee[1,7,*]

[1]Department of Earth System Sciences, Yonsei University, Seoul 03722, Korea

[2]NanoCenter & Department of Chemical Engineering, University of South Carolina, Columbia, SC 29208, USA

[3]NanoCenter & Department of Chemistry & Biochemistry, University of South Carolina, Columbia, SC 29208, USA

[4]Department of Materials Science and Engineering, Yonsei University, Seoul 03722, Korea

[5]State Key Lab of Superhard Materials & Innovation Center for Computational Physics Methods and softwares, College of Physics, Jilin University, 130012 Changchun, China

[6]International Center of Future Science, Jilin University, 130012 Changchun, China

[7]Center for High Pressure Science and Technology Advanced Research, 201203 Shanghai, China

*Corresponding author: yongjaelee@yonsei.ac.kr (Y. L.)

This PDF file includes:

Supplementary Figures 1 to 4
Supplementary Tables 1 to 3

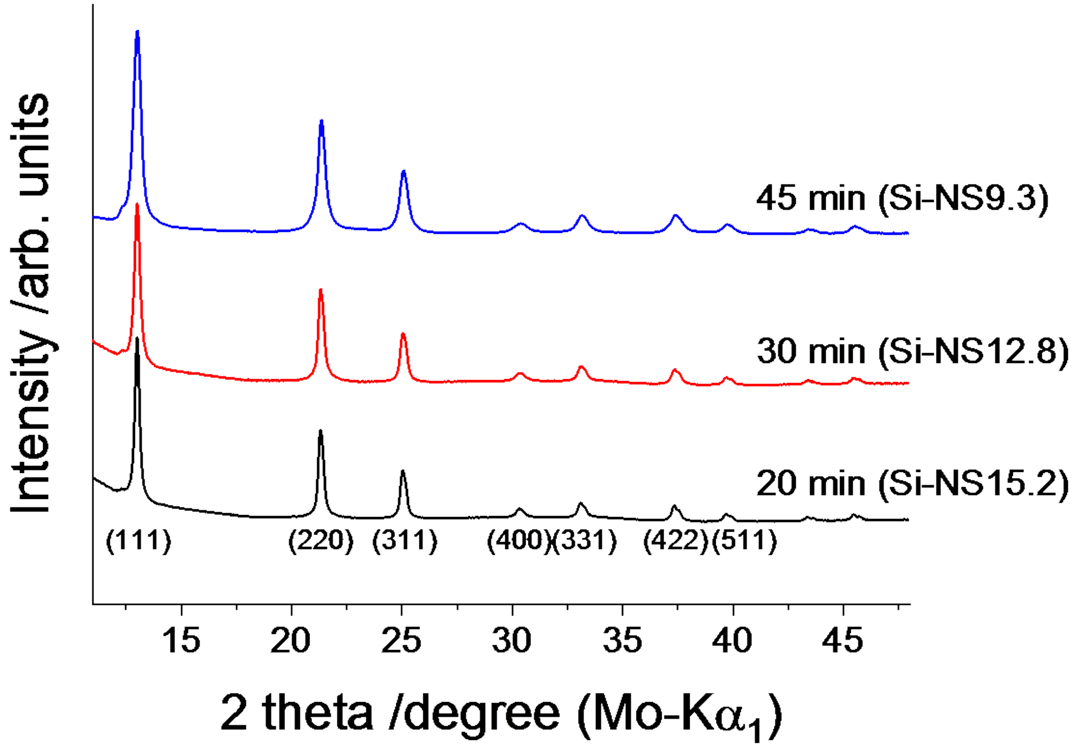

Supplementary Figure 1. X-ray diffraction patterns of Si-NSs at ambient conditions using powder samples inside quartz capillaries (Ø0.5 mm) using Mo-Kα_1 radiation.

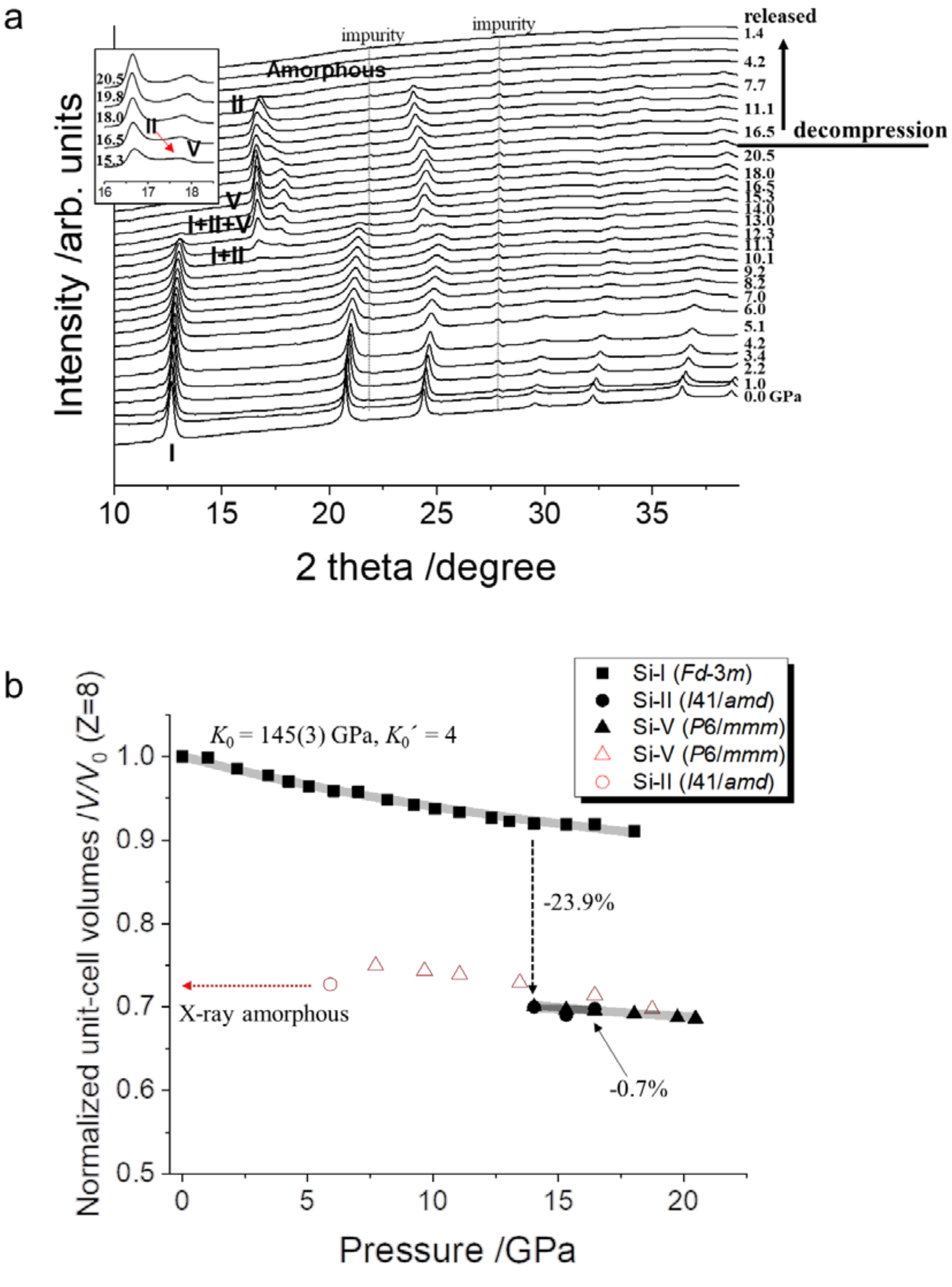

Supplementary Figure 2. **a**, High-pressure X-ray diffraction for Si-NS9.3 subjected to compression and decompression. **b**, Normalized unit cell volumes of phases observed during compression and decompression of Si-NS9.3. X-ray amorphous phases appear after 5.90(10) GPa during compression for Si-NS9.3. The volume was normalized to Z = 8.

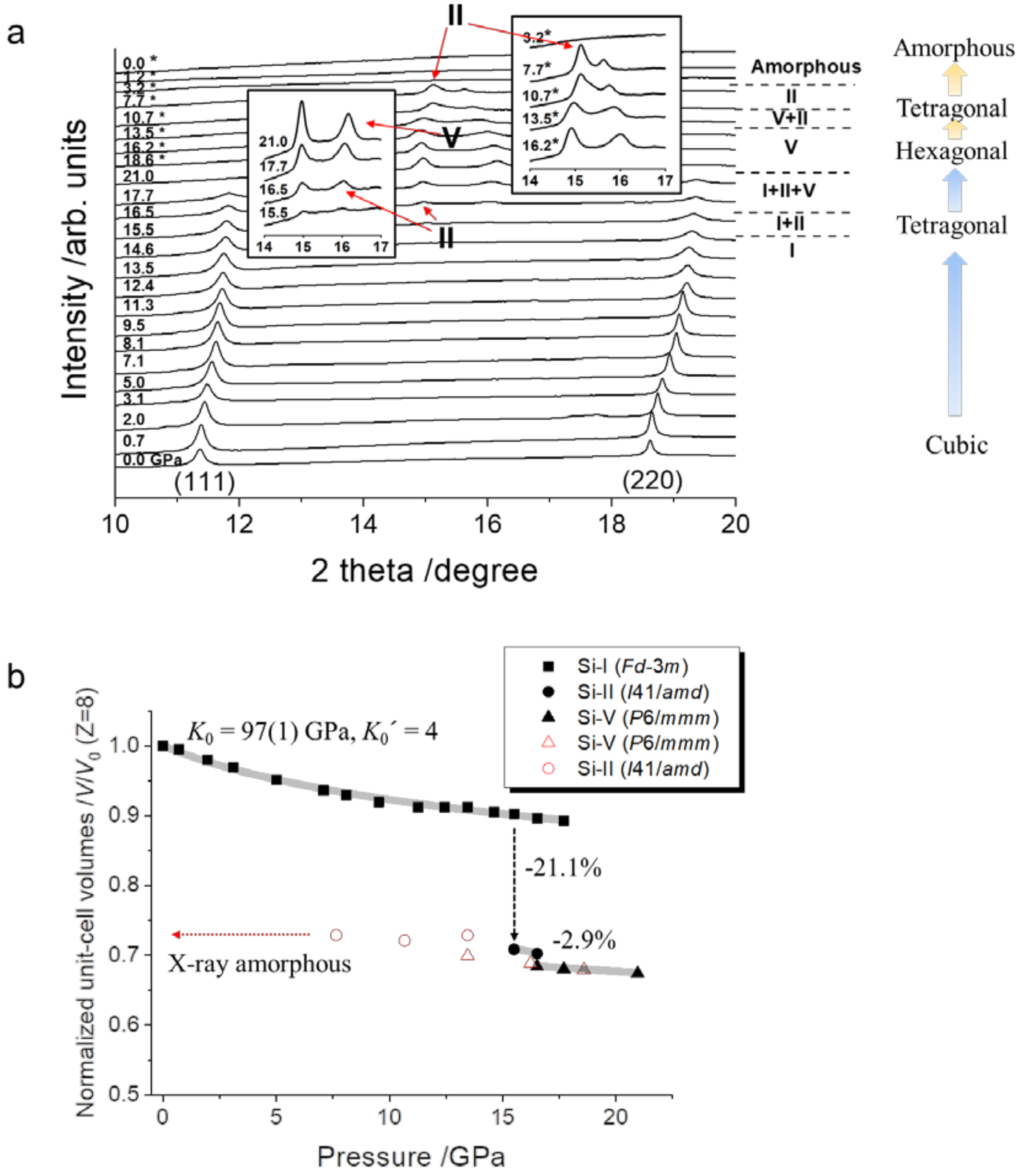

Supplementary Figure 3. **a**, High-pressure X-ray diffraction of Si-NS12.8 subjected to compression and decompression (*). Inset images highlight phase transition region to Si-II and Si-V. **b**, Normalized unit cell volumes of phases observed during compression and decompression cycle of Si-NS12.8. X-ray amorphous phases appear below 7.65(10) GPa during decompression for Si-NS12.8. Unit cell volume was normalized to *Z* = 8.

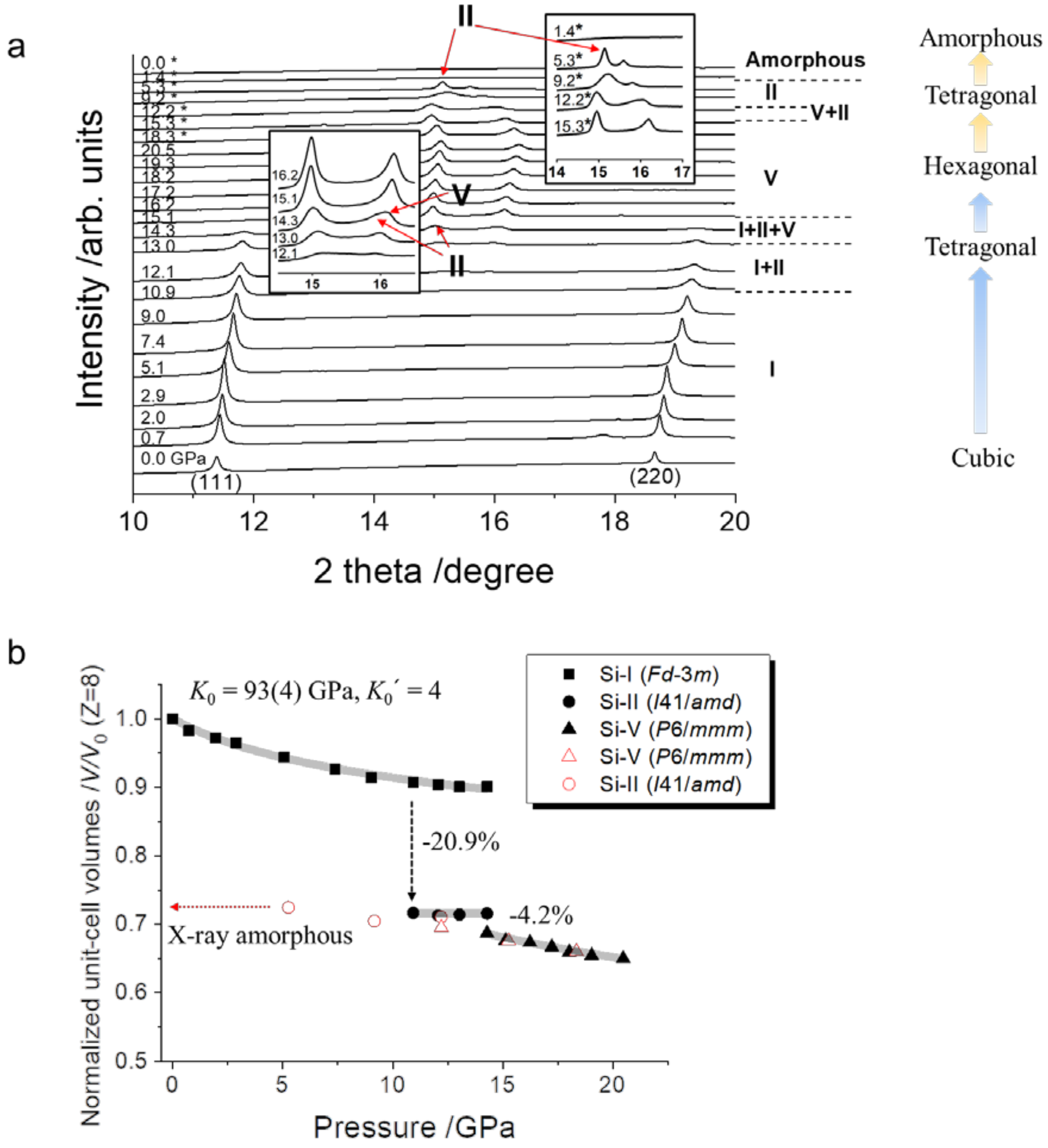

Supplementary Figure 4. **a**, High-pressure X-ray diffraction of Si-NS15.2 during compression and decompression (*). Inset images highlight phase transition regions to Si-II and Si-V. **b**, Normalized unit cell volumes of phases observed during compression and decompression of Si-NS15.2. X-ray amorphous phases appear below 5.26(10) GPa during decompression for Si-NS15.2. Each volume was normalized to $Z = 8$.

Supplementary Table 1. Parameters of Si-NSs and X-ray conditions under ambient conditions

	Si-NS9.3	Si-NS12.8	Si-NS15.2
Composition		Si	
X-ray radiation		Mo-Kα_1 (λ = 0.070932(1) nm)	
Crystal system		*Cubic (diamond structure)*	
Space group		$Fd\bar{3}m$	
a /nm	0.5435(1)	0.5429(1)	0.5411(1)
V /nm^3	0.1606(1)	0.1600(1)	0.1584(1)
FWHM (111)	0.4030(1)	0.2943(1)	0.2475(1)
Average crystallite size /nm*, (111)	9.3(7)	12.8(8)	15.2(4)
Density (X-ray) /gm/cm^3	4.647	4.663	4.710
CVD time	45 min	30 min	20 min
Reference		[1]	

* Crystallite size was calculated using Scherrer equation $D_{(111)} = 0.94\lambda/\beta \cos\theta$[2]

Supplementary Table 2. Bulk moduli (K_0) and lattice parameters of Si-NSs

P /GPa	*a* /nm	*c* /nm	*c/a*	*V* /nm^3	V/V_0	V/V_0 (Z=8)	Phase
**Si-NS9.3**	I (0 - 18.0 GPa); K_0 = 145(3) GPa, K_0' = 4 (fixed)						
0.00 (in DAC)	0.5435(1)			0.1605(1)	1.0000		I
1.01	0.5432(1)			0.1603(1)	0.9987		I
2.18	0.5408(1)			0.1582(1)	0.9854		I
3.42	0.5393(1)			0.1569(1)	0.9773		I
4.24	0.5380(1)			0.1557(1)	0.9700		I
5.05	0.5369(1)			0.1548(1)	0.9644		I
6.04	0.5359(1)			0.1539(1)	0.9587		I
7.01	0.5357(1)			0.1537(1)	0.9577		I
8.17	0.5339(1)			0.1522(1)	0.9482		I
9.24	0.5328(1)			0.1513(1)	0.9422		I
10.08	0.5319(1)			0.1505(1)	0.9377		I
11.06	0.5311(1)			0.1498(1)	0.9334		I
12.33	0.5298(1)			0.1487(1)	0.9265		I
13.04	0.5290(1)			0.1481(1)	0.9224		I
	**			**	**	**	II
14.03	0.5285(1)			0.1476(1)	0.9195		I
	0.4653(1)	0.2595(5)	0.5576(5)	0.0562(1)	1.0000	0.6999	II
	0.2609(1)	0.2384(1)	0.9139(1)	0.0141(1)	1.0000	0.7003	V
15.31	0.5283(1)			0.1475(1)	0.9188		I
	0.4644(1)	0.2569(1)	0.5533(1)	0.0554(2)	0.9862	0.6902	II
	0.2612(1)	0.2365(1)	0.9054(1)	0.0140(1)	0.9948	0.6967	V
16.45	0.5283(6)			0.1475(5)	0.9188		I
	0.4666(1)	0.2569(1)	0.5513(1)	0.0560(10)	0.9972	0.6979	II
	0.2597(1)	0.2390(1)	0.9206(1)	0.0140(1)	0.9932	0.6956	V
18.03	0.5268(6)			0.1462(5)	0.9106		I
	0.2590(1)	0.2389(1)	0.9266(1)	0.0139(1)	0.9878	0.6918	V
19.75	0.2579(1)	0.2393(1)	0.9278(1)	0.0138(1)	0.9811	0.6871	V
20.47	0.2577(1)	0.2393(1)	0.9285(1)	0.0138(1)	0.9796	0.6861	V
18.74 *	0.2595(1)	0.2398(1)	0.9239(1)	0.0140(1)	0.9954	0.6972	V
16.45 *	0.2631(1)	0.2390(1)	0.9085(1)	0.0143(1)	1.0197	0.7141	V
13.46 *	0.2657(1)	0.2393(1)	0.9007(1)	0.0146(1)	1.0522	0.7390	V
11.06 *	0.2675(1)	0.2392(1)	0.8943(1)	0.0148(1)	1.0552	0.7390	V
9.66 *	0.2683(1)	0.2392(1)	0.8913(1)	0.0149(1)	1.0614	0.7434	V
7.71 *	0.2703(1)	0.2376(1)	0.8792(1)	0.0150(1)	1.0700	0.7493	V
5.90 *	0.4738(1)	0.2560(1)	0.5487(2)	0.0584(1)	1.0390	0.7272	II
4.15 *							amorphous
2.59 *							amorphous
1.36 *							amorphous
0.00 *							amorphous
**Si-NS12.8**	I (0 - 17.7 GPa); K_0 = 97(1) GPa, K_0' = 4 (fixed)						
0.00 (in DAC)	0.5430(1)			0.1601(1)	1.0000		I ($Fd\bar{3}m$)
0.70	0.5422(1)			0.1594(1)	0.9954		I
1.97	0.5395(1)			0.1570(1)	0.9804		I
3.10	0.5374(1)			0.1552(1)	0.9694		I
5.01	0.5341(1)			0.1524(1)	0.9517		I
7.09	0.5312(1)			0.1499(1)	0.9360		I
8.09	0.5298(1)			0.1487(1)	0.9291		I
9.53	0.5281(1)			0.1473(4)	0.9199		I
11.27	0.5266(1)			0.1461(1)	0.9124		I
12.43	0.5266(1)			0.1460(1)	0.9121		I

13.45	0.5266(1)			0.1460(1)	0.9121		I
14.61	0.5253(1)			0.1450(1)	0.9055		I
15.50	0.5248(2)			0.1445(2)	0.9028		I
	0.4481(1)	0.2825(1)	0.6305(1)	0.0567(1)	1.0000	0.7086	II (*I*41/*amd*)
16.52	0.5236(1)			0.1435(1)	0.8965		I
	0.4463(1)	0.2823(1)	0.6325(1)	0.0562(1)	0.9914	0.7025	II
	0.2573(1)	0.2387(1)	0.9278(1)	0.0137(1)	1.0000	0.6840	V (*P*6/*mmm*)
17.70	0.5228(1)			0.1429(1)	0.8923		I
	0.2567(1)	0.2386(1)	0.9298(1)	0.0136(1)	0.9946	0.6802	V
20.96	0.2554(1)	0.2388(1)	0.9352(1)	0.0135(1)	0.9856	0.6740	V
18.59 *	0.2561(1)	0.2393(1)	0.9342(1)	0.0136(1)	0.9933	0.6799	V
16.23 *	0.2577(1)	0.2395(1)	0.9297(1)	0.0138(1)	1.0062	0.6882	V
13.45 *	0.2602(1)	0.2386(1)	0.9170(1)	0.0140(1)	1.0218	0.6988	V
	0.4776(1)	0.2558(1)		0.0583(1)	1.0283	0.7287	II
10.67 *	0.4723(1)	0.2588(1)		0.0577(1)	1.0177	0.7211	II
7.65 *	0.4724(1)	0.2615(1)		0.0583(1)	1.0285	0.7288	II
3.24 *							amorphous
1.22 *							amorphous
0.00 *							amorphous
**Si-NS15.2**	I (0 - 14.3 GPa); K_0 = 93(4) GPa, K_0' = 4 (fixed)						
0.00 (in DAC)	0.5429(1)			0.1560(1)	1.0000		I ($Fd\bar{3}m$)
0.74	0.5398(1)			0.1573(1)	0.9831		I
1.95	0.5378(1)			0.1555(1)	0.9722		I
2.89	0.5364(1)			0.1543(1)	0.9648		I
5.06	0.5325(1)			0.1510(1)	0.9438		I
7.38	0.5293(1)			0.1482(1)	0.9267		I
9.02	0.5268(1)			0.1462(1)	0.9141		I
10.93	0.5255(1)			0.1451(1)	0.9072		I
	0.4695(6)	0.2600(4)	0.5538(6)	0.0573(2)	1.0000	0.7166	II (*I*41/*amd*)
12.06	0.5249(2)			0.1446(2)	0.9042		I
	0.4703(4)	0.2579(5)	0.5485(5)	0.0570(2)	0.9951	0.7131	II
13.03	0.5242(9)			0.1440(7)	0.9004		I
	0.4736(1)	0.2547(1)	0.5377(1)	0.0571(1)	0.9966	0.7142	II
14.28	0.5231(1)			0.1432(1)	0.9004		I
	0.4763(1)	0.2524(1)	0.5299(1)	0.0573(1)	0.9988	0.7158	II
	0.2585(2)	0.2374(2)	0.9181(2)	0.0137(1)	1.0000	0.6872	V (*P*6/*mmm*)
15.12	0.2558(1)	0.2387(1)	0.9332(1)	0.0135(1)	0.9842	0.6763	V
16.23	0.2553(1)	0.2386(1)	0.9347(1)	0.0135(1)	0.9803	0.6736	V
17.22	0.2543(1)	0.2382(1)	0.9368(1)	0.0133(1)	0.9708	0.6671	V
18.02	0.2533(1)	0.2375(1)	0.9379(1)	0.0132(1)	0.9602	0.6598	V
19.03	0.2525(1)	0.2371(1)	0.9390(1)	0.0131(1)	0.9527	0.6547	V
20.46	0.2518(1)	0.2367(1)	0.9401(1)	0.0130(1)	0.9461	0.6501	V
18.34 *	0.2532(1)	0.2378(1)	0.9391(1)	0.0132(1)	0.9607	0.6602	V
15.26 *	0.2555(1)	0.2391(1)	0.9359(1)	0.0135(1)	0.9837	0.6760	V
12.20 *	0.2592(1)	0.2389(1)	0.9215(1)	0.0139(1)	1.0114	0.6950	V
	0.4490(1)	0.2821(1)	0.6283(1)	0.0569(1)	0.9922	0.7110	II
9.16 *	0.4529(1)	0.2748(1)	0.6069(1)	0.0564(1)	0.9834	0.7047	II
5.26 *	0.4587(1)	0.2755(1)	0.6006(1)	0.0580(1)	1.0111	0.7246	II
1.41 *							amorphous
0.00 *							amorphous

* On release process

Structure references: I[1], II[3], V[3].

** Unit-cell parameter cannot be calculated

Supplementary Table 3. Comparison of bulk modulus with previous studies

K_0 /GPa	K_0'	Pressure range /GPa	Crystallite type	Reference
100.8	4.7	0.0 - 4.0	Bulk	Vaidya *et al*. (1972)[4]
97.84(2)	4.11	0.0 - 8.0	Bulk	George (1997)[5]
99	3.2	*ab initio* method	Bulk	Yin and Cohen (1982)[6]
97.8	4.1	0.0 - 11	Bulk	Pandya *et al*. (2010)[7]
145(3)	4	0.0 - 18.0	Nanosheet (9.3 nm)	This study (Si-NS9.3)
122(7)	8(2)	0.0 - 18.0	Nanosheet (9.3 nm)	This study (Si-NS9.3)
97(1)	4	0.0 - 17.7	Nanosheet (12.8 nm)	This study (Si-NS12.8)
70(8)	16(4)	0.0 - 17.7	Nanosheet (12.8 nm)	This study (Si-NS12.8)
93(4)	4	0.0 - 14.3	Nanosheet (15.2 nm)	This study (Si-NS15.2)
54(5)	14(2)	0.0 - 14.3	Nanosheet (15.2 nm)	This study (Si-NS15.2)

Supplementary References

1. Wyckoff, R. W. G. Crystal structures: second edition. *John Wiley* **1**, 7-83 (1963).
2. Scherrer, P. Bestimmung der grosse und der inneren struktur von kolloidteilchen mittels Rontgenstrahlen. *Göttinger Nachrichten Gesell.* **2**, 98 (1918).
3. McMahon, M. I. & Nelmes, R. J. New high-pressure phase of Si. *Phys. Rev. B* **47**, 8337-8340 (1993).
4. Vaidya, S. N. & Kennedy, G. C. Compressibility of 22 elemental solids to 45 kbar. *J. Phys. Chem. Solids* **33**, 1377-1389 (1972).
5. George, A. Properties of Crystalline Silicon: Elastic constants and moduli of diamond cubic Si. *An INSPEC publication* **20**, 98-103 (1997).
6. Yin, M. T. & Cohen, M. L. Theory of static structural properties, crystal stability, and phase transformations: Application to Si and Ge. *Phys. Rev. B* **26**, 5668-5687 (1982).
7. Pandya, T. C., Thakar, N. A. & Bhatt, A. D. Analysis of equations of state and temperature dependence of thermal expansivity and bulk modulus for silicon. *J. Phys. Conf. Ser.* **377**, 012097 (2012).
